# Key Elements of mHealth Interventions to Successfully Increase Physical Activity: Meta-Regression

**DOI:** 10.2196/10076

**Published:** 2018-11-12

**Authors:** Lisa V Eckerstorfer, Norbert K Tanzer, Claudia Vogrincic-Haselbacher, Gayannee Kedia, Hilmar Brohmer, Isabelle Dinslaken, Katja Corcoran

**Affiliations:** 1 Institute of Psychology University of Graz Graz Austria; 2 BioTechMed-Graz Graz Austria

**Keywords:** exercise, physical activity, mHealth, behavior change, meta-analysis, meta-regression

## Abstract

**Background:**

Mobile technology gives researchers unimagined opportunities to design new interventions to increase physical activity. Unfortunately, it is still unclear which elements are useful to initiate and maintain behavior change.

**Objective:**

In this meta-analysis, we investigated randomized controlled trials of physical activity interventions that were delivered via mobile phone. We analyzed which elements contributed to intervention success.

**Methods:**

After searching four databases and science networks for eligible studies, we entered 50 studies with N=5997 participants into a random-effects meta-analysis, controlling for baseline group differences. We also calculated meta-regressions with the most frequently used behavior change techniques (behavioral goals, general information, self-monitoring, information on where and when, and instructions on how to) as moderators.

**Results:**

We found a small overall effect of the Hedges *g*=0.29, (95% CI 0.20 to 0.37) which reduced to *g*=0.22 after correcting for publication bias. In the moderator analyses, behavioral goals and self-monitoring each led to more intervention success. Interventions that used neither behavioral goals nor self-monitoring had a negligible effect of *g*=0.01, whereas utilizing either technique increased effectiveness by Δ*g*=0.31, but combining them did not provide additional benefits (Δ*g*=0.36).

**Conclusions:**

Overall, mHealth interventions to increase physical activity have a small to moderate effect. However, including behavioral goals or self-monitoring can lead to greater intervention success. More research is needed to look at more behavior change techniques and their interactions. Reporting interventions in trial registrations and articles need to be structured and thorough to gain accurate insights. This can be achieved by basing the design or reporting of interventions on taxonomies of behavior change.

## Introduction

People spend much time with their mobile phones (on average 2 hours and 27 minutes per day using apps and the Web [1]) and too little time being physically active [2,3], even though physical activity is beneficial for both body [4-6] and mind [6,7]. Recently, researchers have been looking for ways to use mobile phones to increase physical activity. These mobile health (mHealth) interventions have only been possible in recent years with the upsurge of mobile communication devices [8-11].

For researchers who strive to change health-related behavior, mHealth shows excellent promise for building life-changing interventions, but their efficacy is currently uncertain [12]. In the domain of physical activity, mHealth interventions yield small to moderate effects [13]. To date, researchers have not been able to pinpoint contextual elements that lead to greater intervention success in increasing physical activity [13-19]. Indeed, a wide range of interventions are implemented that often overlap with each other. However, it is still unclear which basic principles and strategies lead to efficient changes. Tomlinson and colleagues [20] pointed out that there should be a joint effort among researchers to find and refine strategies for successful mHealth interventions. Hence, we know that mHealth interventions have great potential, but we need to establish how to maximize their efficacy. This meta-analysis study aims to identify content-related elements that predict intervention efficacy. The resulting knowledge is crucial for creating new interventions and guidelines.

One way to describe the content of interventions is to identify the behavior change techniques (BCTs) on which they rely. These are theory-based methods to change psychological determinants of behavior, such as agreeing on a behavioral contract or facilitating social comparison. Until now, it is unclear which BCTs contribute more to mHealth intervention success than others. This is mostly because until recently, only a limited number of studies have been available. Previous reviews and meta-analyses identified n=11 [15], n=19 [13], and n=18 [19] studies. These pools of studies only provide enough power to test two or three moderators in a meta-regression [21], so there was no feasible way to test the influence of each BCT—first, because there is a considerable number of BCTs and second, because some BCTs are seldom used or not used at all [13,22].

In the present meta-analysis, we decided to use a taxonomy by Michie and colleagues [23], which—in contrast to more general taxonomies for behavior change [24]—focuses on BCTs for diet and physical activity interventions. This taxonomy contains 40 BCTs, all of which we coded, but we only tested the influence of the 5 most frequently used ones in order to retain sufficient statistical power. In our sample, these are (1) behavioral goals, (2) general information, (3) self-monitoring, (4) information on where and when, and (5) instructions on how to. One change we wanted to make in comparison to previous work is to control for group differences at baseline. Even though we only used randomized controlled trials (RCTs), we expected to find a high number of feasibility and pilot tests with small sample sizes, in which not all the advantages of randomization can unfold properly. Our main research question is which of the 5 most frequently used BCTs have the potential to increase the efficacy of mobile phone-delivered physical activity interventions?

## Methods

### Searching for Studies

To find suitable studies, we searched Google scholar and 4 databases: PubMed, PsycINFO, ScienceDirect, and the ISI Web of Knowledge using search terms related to physical activity, mHealth and study design. An example for a search syntax is *([randomized controlled trial OR RCT OR randomised controlled trial OR clinical trial] AND [mobile phone OR smartphone OR mobile app OR mHealth] AND [exercise OR physical fitness OR physical activity])*. We did not restrict year of publication, but we did restrict language to English or German. Furthermore, we searched reference lists of published reviews on the topic [14,15,19] and posted invitations to research communities in health psychology, sports psychology, social psychology, and sports science to contribute relevant studies.

### Selecting Studies

**Figure 1:**
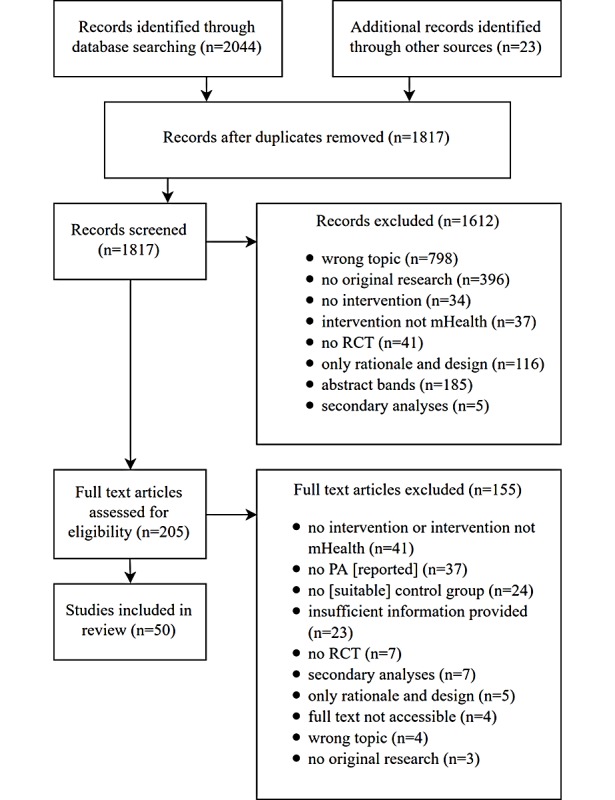
Flow diagram of the study selection process. RCT: randomized controlled trial; PA: physical activity; mHealth (mobile health).

We did not restrict inclusion to specific populations. Instead, we accepted all studies that targeted physical activity—be it for healthy populations, sick populations, during pregnancy, or for children (Textbox 1). The general flow of study selection is presented in Figure 1.

We identified a total of 2067 studies. After removing duplicates, we screened the remaining 1817 records for eligibility according to their title and abstract. We assessed the full text of 205 studies and from these, 50 met the inclusion criteria [25-74]. When we screened titles and abstracts, we excluded poorly fitting records hierarchically. First, we reviewed content fit, and then we checked whether the research was original. Following that, we assessed whether there was an intervention and whether the intervention was delivered via mobile phone—and so on. In the second screening, we checked those points again—but more thoroughly—and we also assessed more complex questions, such as the suitability of the control group or whether there was enough information to compute effect sizes of group differences at baseline and the end of the intervention.

**Study inclusion and exclusion criteria.**

**Inclusion criteria**

- The intervention was automatically delivered via mobile phone by either an app or texting
- The intervention targeted an increase in physical activity
- There was a control group, which was more passive than the intervention group and did not have personal communications with medical staff or researchers instead of receiving the mHealth intervention
- Allocation to experimental and control groups was randomized, though we accepted stratification (eg, by gender)
- At least one of the outcomes measured actual physical activity (via electronic trackers or self-reported) or assessed objective indicators of physical fitness (eg, peak oxygen intake)

**Exclusion criteria**

- Study designs were nonexperimental (eg, observational studies, reports and comments, case studies)
- The data necessary to calculate an effect size was not available

### Outcome Measures and Effect Size

In the present meta-analysis, we wanted to investigate how selected interventions influence physical activity; hence we focused on physical activity as outcomes. There are 2 main methods to measure physical activity: having people self-report their physical activity and electronically tracking people’s movement. Self-reported and tracked physical activity generally correlate poorly or moderately with each other, but with a range from *r*=–.71 to .96 [75]. To check whether the way in which physical activity was measured in our studies impacts our main results, we investigated the influence of physical activity measurement on intervention success by including the type of outcome (tracked versus self-reported versus both) as a moderator in our design.

Since our pool of studies includes many pilot and feasibility tests, we decided to account for their small sample size by using the Hedges *g* to measure effect size. This is principally the same measurement as the Cohen *d*, but it uses a correction factor for small sample sizes [76]. The interpretation of the Hedges *g* follows the same rule of thumb that applies to the Cohen *d*:|*g* |=0.20 for a small effect, |*g* |=0.50 for a medium effect, and |*g* |=0.80 for a large effect.

### Review Procedure and Moderators

The extracted studies were initially screened for eligibility and then articles were classified according to title and abstract. The categories are depicted in the list of “Records excluded” (Figure 1). The procedure was conducted hierarchically, first making sure the topic fitted, then checking the originality of the data (ie, if there was any data collected), implementation of an intervention, mHealth focus of that intervention, and so on. A consensus was reached through discussion. Information was then extracted from the articles and protocols into a Microsoft Excel spreadsheet. Of the 205 studies of which the full text was assessed for eligibility, 96 were coded twice to uphold the same rating standards. Again, a consensus was reached through discussion in several evaluation sessions.

To estimate study quality, we used the Effective Public Health Practice Project (EPHPP) Quality Assessment Tool [77]. The EPHPP tool has 6 sections, which are aggregated to a final grade on a 3-point scale. Low study quality is a known issue in electronic interventions [78,79], but we did not exclude those studies. Instead, we checked whether the study quality moderates intervention efficacy, because a correspondence can be a sign of publication bias.

Further, some of the studies we included did not (only) target physical activity, but health or weight, with an increase of physical activity being one of multiple intervention goals. We expected that mHealth interventions directly targeting physical activity rather than health or weight would be more efficient in increasing physical activity. Therefore, we assessed the studies’ main objective as a moderator of intervention success.

### Behavior Change Techniques

To identify the factors of mHealth intervention success, we used a taxonomy of 40 BCTs to code intervention contents [23]. We coded only BCTs that were employed in the intervention group and not in the control group. If a BCT was used in both groups, we counted it as absent from the intervention. Since we only expected to collect a sample of studies large enough to test 5 moderators (ie, around 25 to 50 studies), we decided to test the five most frequently used BCTs as moderators. The BCTs we tested were (1) behavioral goals, (2) general information, (3) self-monitoring, (4) information on where and when, and (5) instructions on how to.

### Meta-Analysis and Meta-Regression

To assure quality in our meta-analysis, we consulted with experts, and followed the recommendations in Borenstein and colleagues [76], and consulted a quality assessment tool for meta-analyses [80]. All statistical analyses in the present study were conducted in R, version 3.4.2 [81] with the additional packages robumeta [82], metafor [83], MAd [84], tidyverse [85], readxl [86], compute.es [87], stats [81], ggplot2 [88], yarrr [89], and wesanderson [90]. We calculated the effect size (Hedges *g*) for the difference between the intervention and control group before the intervention (baseline) and at the end of the scheduled intervention (postintervention) from means, standard deviations and group size, *P*-values or proportions. To assess whether there were meaningful physical activity group differences before the interventions were administered, we first performed a random-effects meta-analysis with baseline group differences. Then, we correlated baseline group differences with postintervention group differences. Since there was more heterogeneity at baseline than expected by chance alone and baseline group differences were not independent of intervention success, we used baseline group differences as a covariate in further analyses, essentially setting physical activity group differences before the intervention to zero. We also removed outliers at baseline and repeated the overall effect size analyses with this reduced pool of studies.

For the meta-analysis with postintervention group differences and meta-regression we used robumeta, as recommended in a review of meta-analysis packages [91]. This package allows for dependent effect estimation (ie, combining multiple outcomes per study in a correlated or hierarchical fashion) [92]. We coded up to four outcomes per study and aggregated those with a correction for small sample sizes. Since we were not able to predict the correlation between outcomes, we performed sensitivity analyses. Varying the assumed correlation only led to negligible outcome changes in the second decimal place regarding the estimated coefficients and tau-squared (*τ²*). For our main analyses, we first ran a random effects null-model to determine overall intervention success. Then, we checked— separately for each moderator—whether study quality, the intervention’s main objective, physical activity tracking or any of the most frequently used BCTs were associated with greater intervention success. For each BCT, we compared interventions that used it with interventions that did not use it. In an exploratory fashion, we also investigated a combination of the two most efficient BCTs. We did not run any more moderator analyses to avoid exceeding the power of our pool of 50 studies.

### Publication Bias

To evaluate a possible publication bias in the field, we created a funnel plot and performed the Egger asymmetry test [93] as recommended in [76]. We also performed a trim and fill analysis [94] and the Orwin Failsafe-N test to assess whether the effect might be an artifact of bias [95]. Due to a large proportion of feasibility and pilot trials with small sample sizes in our pool of studies, we decided against restricting the analysis to large samples.

## Results

### Selected Studies

For our meta-analysis, we had a final pool of 50 studies with N=5997 people. The mean age of included samples was 40.6 (SD 16.7), and on average there were 62.7% women in each study (SD 29.2). We found that 40/50 (80%) studies were of good quality, 5 of the 50 (10%) were of moderate quality, and the remaining 10% (5/50) were of poor quality. Twenty-nine of the 50 (58%) studies targeted physical activity, 13/50 (26%) targeted health and 16% (8/50) targeted weight. Furthermore, 20 of the 50 (40%) studies used self-reported measures of physical activity, 17/50 (34%) tracked physical activity, and 13/50 (26%) studies used both methods. We coded a maximum of 4 outcomes per study (*M*=1.76), which led to 87 physical activity-related outcomes across the pool of 50 studies. Sample sizes and group differences at baseline as well as after the intervention are provided in Table 1. A table with more details regarding population, intervention, comparison, and outcomes can be found in Multimedia Appendix 1.

Not all BCTs were used equally often. Therefore, some BCTs are featured in more than half of the studies whereas others are not featured at all. Information for each BCT of the taxonomy is available in Multimedia Appendix 1. We decided to test the five most frequently used BCTs as moderators. As shown in Table 2, there are 18 to 30 studies out of 48 using each of these 5 BCTs. Table 2 also indicates the number of studies in each element in which physical activity was measured as self-reports, electronically tracked, or if both methods were used simultaneously. We ran a chi-square test to determine whether using a certain BCT was confounded by how physical activity was measured. The test did not reveal a significant association between using BCTs and the way in which physical activity was measured (χ²_8_=13.6, *P*=.09). Overall—in 48/50 (96%) studies—235 BCTs were used, which led to a mean of 5 BCTs per study.

### Baseline Group Differences

A random-effects meta-analysis with baseline group differences revealed that the estimated effect *g*=0.05 was not significantly different from zero (95% CI –0.03 to 0.13, *P*=.22), but there was considerable heterogeneity between the studies (*Q* (49)=93.79, *P*<.001) and 43.33% of this heterogeneity was due to true effect sizes rather than sampling variance (*I²*=43.33%). Overall, the variance of true effects was *τ²*=0.03 on a scale of the Hedges *g*.

**Figure 2:**
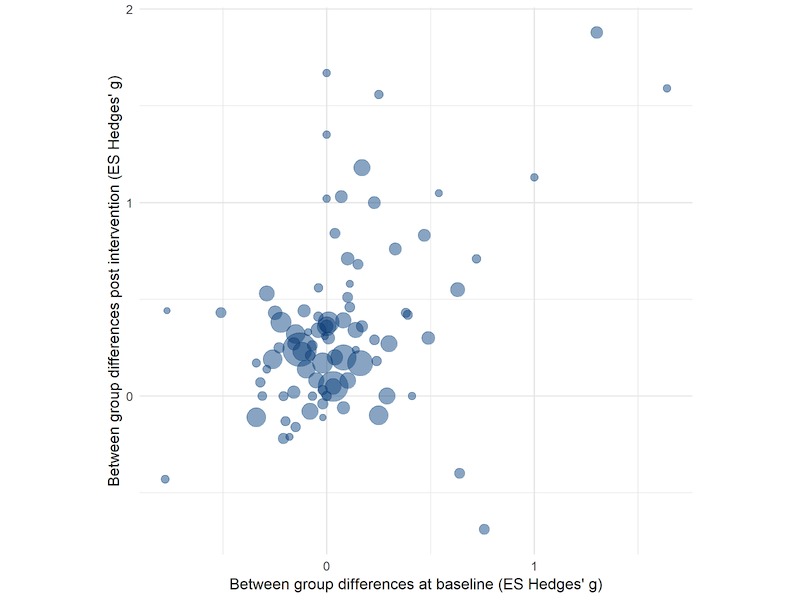
Scatterplot of group differences before and after the intervention for each outcome separately. Point size indicates the number of participants in each study. ES: effect size.

Correlating group differences at baseline with group differences postintervention showed a positive association of *r*=.44 (*P*<.001, see Figure 2). This means that when participants who received the intervention were more physically active at baseline than people in the control group, this advantage was also conveyed to group differences after the intervention. To consider this phenomenon, we took baseline differences into account in 2 separate ways. Firstly, we used baseline group differences as a covariate—setting group differences to zero. Secondly, we removed outcomes with group differences of |*g* |>0.50 at baseline and reran overall effect size analyses with this reduced pool of studies. In the reduced pool, there were 44 studies with 76 outcomes. We present a scatterplot of outcomes at baseline by outcomes postintervention (see Figure 2).

### Point Estimate

**Figure 3:**
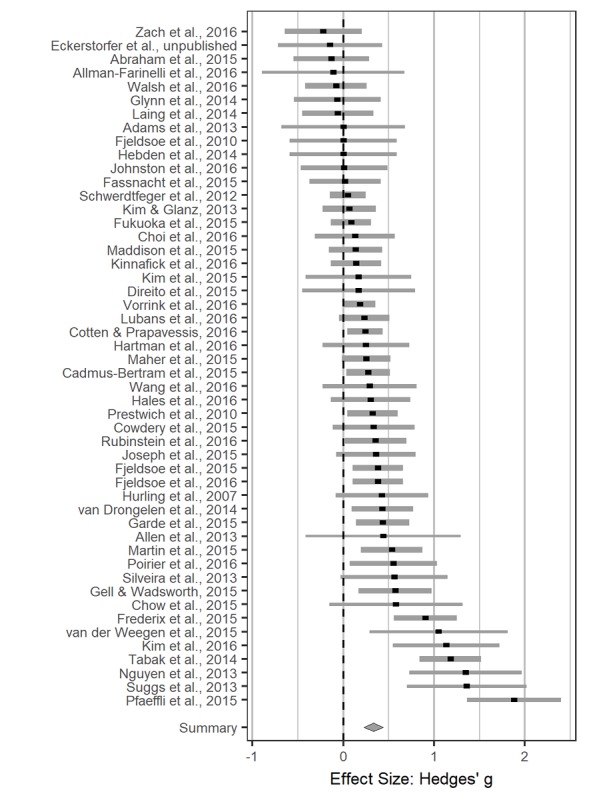
Forest plot for physical activity postintervention. The larger the values, the more active the intervention group was compared to the control group. Horizontal lines depict 95% CI and line thickness indicates each number's impact on the summary effect.

We assumed a correlation of *r*=.80 between outcomes of the same study. We did not have the means to check whether this assumption was valid, so we also performed sensitivity analyses—varying the assumed correlation between 0 and 1—and we did not find meaningful changes in the results. A random effects model for group differences postintervention revealed an estimated effect size of *g*=0.33 (95% CI 0.22 to 0.44, *P*<.001). We show a forest plot of group differences after the intervention is presented (see Figure 3). However, including baseline differences as a covariate in the same analysis reduced the estimated effect size to *g*=0.29 (95% CI 0.20 to 0.37, *P*<.001) with a covariate slope estimate of *g*=0.63 (95% CI 0.11 to 1.14, *P*=.02), meaning that increasing baseline group differences by *g*=1.00 would lead to a greater intervention success of *g*=0.63. True means had a variance of *τ²*=0.06 on a scale of the Hedges *g*, and 61.15% of the observed heterogeneity was due to true differences between studies rather than chance, so we went ahead with the planned moderator analyses. Estimating the overall effect size with a reduced pool of outcomes instead of baseline group differences as covariate painted much the same picture (estimated effect size *g*=0.28, 95% CI 0.19 to 0.38, *P*<.001, *I²*=56.38%, *τ²*=0.05).

### Moderator Analyses

All moderator analyses were conducted using baseline group differences as a covariate. They did not reveal a significant influence of study quality (Δ*g*=0.03, 95% CI –0.15 to 0.22, *P*=.70). In terms of an intervention’s main objective, targeting weight rather than physical activity reduced the success of increasing physical activity, but there were no differences between health and physical activity or health and weight as the main objective (Δ*g_PA versus weight_*=–0.21, 95% CI –0.42 to –0.003, *P*=.047; Δ*g_health versus PA_*=0.13, 95% CI –0.09 to 0.36, *P*=.23; Δ*g_health vs weight_*=–0.08, 95% CI –0.29 to 0.14, *P*=.45). There was no difference between interventions in which people self-reported their physical activity and interventions whereby physical activity was tracked, and those interventions in which both methods were used simultaneously (Δ*g_self-reported versus tracked_*=0.01, 95% CI –0.21 to 0.24, *P*=.90; Δ*g_both versus self-reported_*=–0.11, 95% CI –0.34 to 0.11, *P*=.31; Δ*g_both versus tracked_*=–0.12, 95% CI –0.36 to 0.11, *P*=.27).

**Table 1:** Overview of included studies showing the Hedges *g* effect size of group differences.

Study	N	Baseline, *g*^a^	Postintervention, *g* (95% CI)
Abraham et al 2015 [25]	32	0.41	0.00 (–0.68 to 0.68)
Adams et al 2013 [26]	20	–0.77	0.44 (–0.41 to 1.29)
Allen et al 2013 [27]	23	–0.02	–0.11 (–0.89 to 0.67)
Allman-Farinelli et al 2016 [28]	248	–0.12	0.28 (0.03 to 0.52)
Cadmus-Bertram et al 2015 [29]	51	–0.10	0.13 (–0.31 to 0.57)
Choi et al 2016 [30]	29	0.11	0.58 (–0.15 to 1.31)
Chow et al 2015 [31]	710	–0.13	0.24 (0.04 to 0.44)
Cotten and Prapavessis 2016 [32]	56	0.02	0.34 (–0.11 to 0.78)
Cowdery et al 2015 [33]	39	–0.34	0.17 (–0.45 to 0.79)
Direito et al 2015 [34]	34	–0.54	–0.14 (–0.72 to 0.43)
Eckerstorfer et al (unpublished data)	95	–0.16	0.02 (–0.37 to 0.41)
Fassnacht et al 2015 [35]	45	–0.31	0.00 (–0.59 to 0.59)
Fjeldsoe et al 2016 [36]	216	–0.09	0.09 (0.56 to 1.25)
Fjeldsoe et al 2015 [73]	266	0.01	0.38 (0.09 to 0.68)
Fjeldsoe et al 2010 [37]	88	0.28	0.91 (–0.13 to 0.30)
Frederix et al 2015 [38]	139	–0.17	0.44 (0.14 to 0.73)
Fukuoka et al 2015 [39]	61	0.06	0.57 (0.17 to 0.97)
Garde et al 2015 [40]	47	–0.21	–0.07 (–0.55 to 0.42)
Gell and Wadsworth 2015 [41]	87	0.01	0.30 (–0.14 to 0.74)
Glynn et al 2014 [42]	66	–0.23	0.25 (–0.23 to 0.73)
Hales et al 2016 [43]	43	–0.07	0.00 (–0.59 to 0.59)
Hartman et al 2016 [44]	50	0.39	0.43 (–0.08 to 0.93)
Hebden et al 2014 [45]	51	0.05	0.01 (–0.47 to 0.49)
Hurling et al 2007 [46]	77	0.17	0.36 (–0.08 to 0.80)
Johnston et al 2016 [47]	151	0.07	0.07 (–0.23 to 0.36)
Joseph et al 2015 [48]	28	–0.04	0.17 (–0.41 to 0.75)
Kim and Glanz 2013 [49]	41	0.49	1.14 (0.55 to 1.72)
Kim et al 2015 [50]	196	–0.10	0.14 (–0.14 to 0.42)
Kim et al 2016 [51]	95	0.08	–0.06 (–0.45 to 0.33)
Kinnafick et al 2016 [52]	65	–0.12	–0.13 (–0.55 to 0.29)
Laing et al 2014 [53]	211	–0.12	0.23 (–0.05 to 0.51)
Lubans et al 2016 [54]	157	0.30	0.14 (–0.16 to 0.43)
Maddison et al 2015 [55]	143	0.05	0.25 (–0.02 to 0.52)
Maher et al 2015 [56]	98	0.05	0.54 (0.20 to 0.87)
Martin et al 2015 [57]	32	0.00	1.35 (0.73 to 1.97)
Nguyen et al 2013 [58]	84	1.30	1.88 (1.36 to 2.40)
Pfaeffli et al 2015 [59]	123	0.63	0.55 (0.07 to 1.03)
Poirier et al 2016 [60]	217	–0.15	0.32 (0.04 to 0.60)
Prestwich et al 2010 [61]	94	–0.14	0.36 (0.02 to 0.69)
Rubinstein et al 2016 [62]	553	0.03	0.05 (–0.15 to 0.25)
Schwerdtfeger et al 2012 [63]	43	–0.04	0.56 (–0.03 to 1.15)
Silveira et al 2013 [64]	31	1.32	1.36 (0.70 to 2.02)
Suggs et al 2013 [65]	158	0.17	1.18 (0.84 to 1.52)
Tabak et al 2014 [66]	29	0.54	1.05 (0.29 to 1.81)
van der Weegen et al 2015 [67]	117	–0.25	0.43 (0.09 to 0.77)
van Drongelen et al 2014 [68]	390	0.12	0.19 (0.02 to 0.35)
Vorrink et al 2016 [69]	157	–0.08	–0.08 (–0.42 to 0.26)
Walsh et al 2016 [70]	55	0.23	0.29 (–0.23 to 0.81)
Wang et al 2016 [71]	59	0.30	–0.22 (–0.64 to 0.20)
Zach et al 2016 [72]	100	0.49	0.30 (–0.09 to 0.69)

^a^Hedges *g* refers to the effect size of group differences, where larger values indicate more physical activity of the intervention group compared to the control group.

**Table 2:** The number of studies using each tested behavior change technique overall and split for the way in which physical activity was measured.

Behavior change technique	N	Self-reported, n, (%)	Tracked, n (%)	Both, n (%)
Behavioral goals	30	10 (33)	8 (27)	12 (40)
Self-monitoring	26	5 (19)	8 (31)	13 (50)
General information	24	9 (38)	11 (46)	4 (17)
Information on where and when	19	6 (32)	11 (58)	2 (11)
Instructions on how to	18	4 (22)	8 (44)	6 (33)

**Figure 4:**
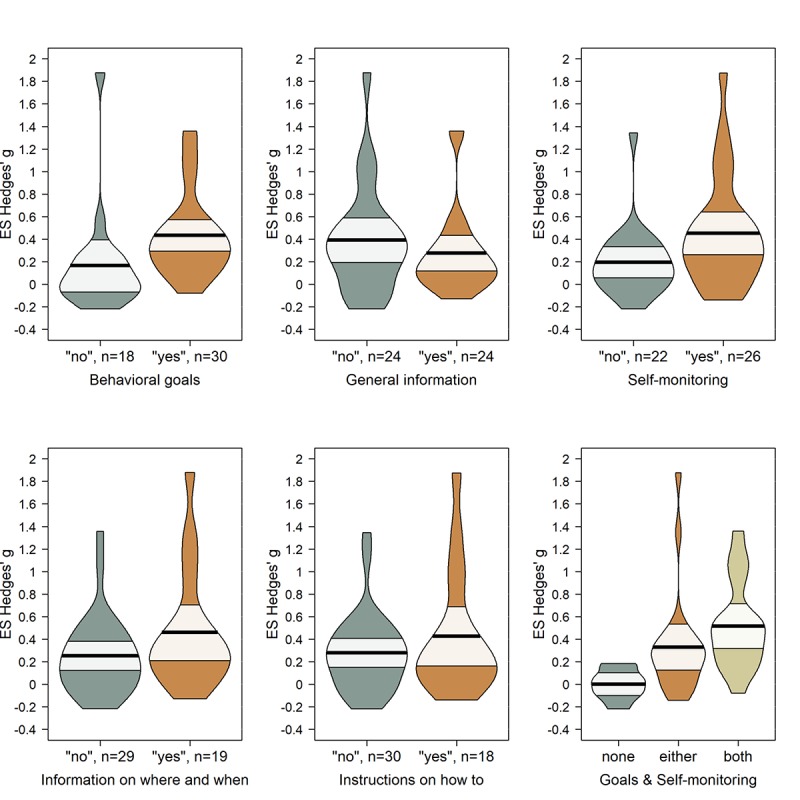
Differences in intervention efficacy depending on the use of the 5 most common behavior change techniques (BCTs). In each panel, “no” means that the BCT was not used and “yes” means that the BCT was used. The “n” next to “yes” and “no” indicates the number of studies in each group. The black line shows the mean intervention efficacy with a 95% CI in white. The curved areas depict density of data points (ie, fine-grained vertical histograms) for all included studies. The last panel shows intervention efficacy depending on a combination of behavioral goals and self-monitoring (n(none)=9, n(either)=22, n(both)=17). These depictions are not controlled for baseline group differences.

Also, we analyzed the effect of the five most frequently used BCTs. Two studies could not be coded for BCTs because they used very diverse interventions and from the text, it was not clear which participants received which intervention components. Thus, we conducted the analyses with a pool of 48 studies, using 84 outcomes. The moderator analyses are visually presented (see Figure 4). Using the BCTs behavioral goals and self-monitoring led to greater intervention success, but the other 3 tested BCTs (ie, general information, information on where and when, and instructions on how to) were not associated with greater intervention success (behavioral goals: Δ*g*=0.20, 95% CI 0.02 to 0.38, *P*=.03; general information: Δ*g*=–0.16, 95% CI –0.33 to 0.01, *P*=.07; self-monitoring: Δ*g*=0.17 95% CI 0.01 to 0.34, *P*=.04; information on where and when: Δ*g*=0.11, 95% CI –0.07 to 0.30, *P*=.22; instructions on how to: Δ*g*=0.01, 95% CI –0.17 to 0.19, *P*=.88). We decided to explore the BCTs behavioral goals and self-monitoring further by combining them in a single moderator model and checking for additive effects. This analysis revealed that if neither behavioral goals nor self-monitoring are used this is associated with a lower intervention efficacy compared with the use of either or both BCTs (Δ*g_none versus either_*=0.31, 95% CI 0.13 to 0.49, *P*=.002; Δ*g_none versus both_*=0.36, 95% CI 0.15 to 0.56, *P*=.002; Δ*g_either versus both_*=0.05, 95% CI –0.19 to 0.29, *P*=.67). More precisely, interventions using neither goals nor rewards have an estimated effect of *g*=0.01, while interventions in which goals and rewards are used simultaneously have an estimated effect of *g*=0.36.

**Figure 5:**
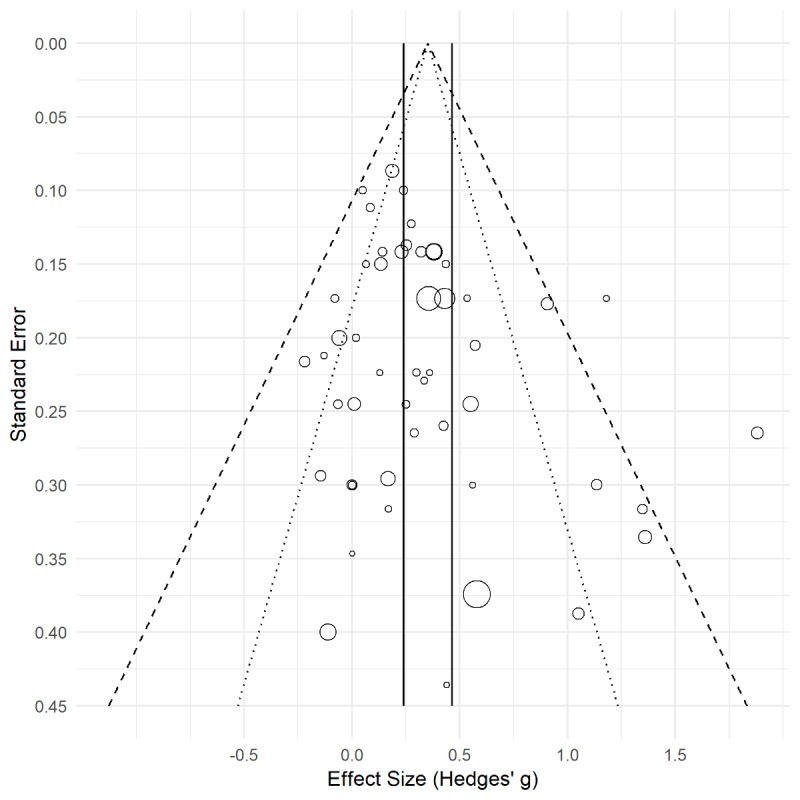
Funnel plot to assess publication bias. Point size indicates the number of participants in each study. The dotted and dashed lines show a 95% and 99% credibility region respectively and the full lines represent a 95% CI for the summary effect.

### Publication Bias

To assess possible publication bias visually, we drew a funnel plot (see Figure 5). On the right side, there were 6 studies outside the 95% credibility region and 2 of those 6 were outside the 99% credibility region. The trim and fill method also suggested that 7 studies are missing on the left side (SE 4.00). These studies might lead to an overestimation of the overall effect. Filling in these “missing” studies led to an adjusted estimated overall effect size of *g*=0.22, meaning that we overestimated by Δ*g*=0.07. The Egger asymmetry test was not significant (*z*=1.53, *P*=.12). Due to its low power in small pools of studies, this nonsignificance does not rule out that no studies are missing. However, the Orwin Failsafe-N test suggests that to obtain a point estimate half the size of the original effect (ie, *g*=0.14), we would need 76 more studies with a nil effect. One hundred and thirty-three studies with a nil effect would be needed to reduce the effect to a negligible effect size of *g*=0.10. Overall, publication bias seems to be acceptably low.

## Discussion

This meta-analysis aimed to identify which BCTs contribute to behavior change in mHealth interventions targeting physical activity. We found an association between heightened efficacy and behavioral goals and the same for self-monitoring. Using either of these techniques pushed the effect from *g*=0.01 to *g*=0.32, but we found no further significant benefit from combining them in an intervention (*g*=0.36). Overall, we found a small to moderate effect of interventions on physical activity, which agrees with another recent meta-analysis of mHealth physical activity interventions [13]. Furthermore, targeting physical activity directly instead of weight can lead to a greater increase of physical activity, but we did not find that the study quality or the tracked versus self-reported data contributed to the intervention efficacy. We also found that neglecting baseline group differences would lead to an overestimation of the efficacy of interventions in this pool of diverse RCTs with many small sample sizes.

To our knowledge, this is the most comprehensive assessment of mHealth interventions to increase physical activity so far. Further, this is the first one to address baseline group differences as covariates and to analyze moderating effects of the most frequently used BCTs while retaining sufficient power. However, 34/50 (68%) of all included studies were published in the years 2015 and 2016. This confirms the rapid growth of the mHealth literature concerned with increasing physical activity. To facilitate sequential meta-analyses and to increase transparency, we are sharing the information we coded and the annotated R script in Multimedia Appendix 2.

### Limitations

There are 2 caveats we would like to address. Firstly, we did not look at actual changes within groups over time because the correlation between baseline and postintervention, which is necessary for this analysis, was generally not reported. Instead, we assessed group differences at baseline and postintervention. Using this approach, an increase of physical activity in the intervention group compared to the control group led us to assume intervention success. However, we also assumed intervention success if physical activity in the control group decreased during the intervention while being stable in the intervention group. Of course, the effects we analyzed might also have been a combination of both (ie, an increase of physical activity in the intervention group and a decrease of physical activity in the control group). Secondly, 4 studies in our pool of 50 (8%) studies used clustered randomization methods (for example by school) [41,54,56,67]. We did not differentiate those studies from fully RCTs, because we did not expect an interaction between the intervention effect and the type of unit randomized (ie, person versus school) [95].

### Future Research

When more studies are available, we will have the statistical power for more sophisticated moderator analyses like meta-CART [96]. Our results suggest that behavioral goals and self-monitoring are especially beneficial, but it remains unclear which other BCTs work well and what interactions there are. Many BCTs from the taxonomy were not used at all, or only very little, and it was hard to code BCTs based on the available information. For the field as a whole it would be beneficial to report intervention content in a more structured way—if possible, based on a BCT taxonomy [24,97] to counteract the current confusion [20], and make our findings more replicable. Furthermore, not all BCTs might work equally well in each context and for each person. However, to assess this question, more research is needed.

In this meta-analysis, we only looked at BCTs, but of course, there is much more to think about when designing successful mHealth interventions. Intervention efficacy does not only depend on BCTs as intervention components, but also on the target population, the intervention design, and duration, as well as the intervention objectives. For example, participants who suffer from illness might be more motivated to use an mHealth intervention because they suffer more than healthy participants. Additionally, healthy participants might already be quite physically active and would therefore not gain a lot from an mHealth intervention targeting motivation to be physically active. Further, BCTs need to be carefully matched with intervention objectives. For example, when an intervention targets capability for physical activity, BCTs related to self-belief might have a greater impact on intervention success than behavioral goals and self-monitoring.

### Conclusion

Despite a small to moderate overall success in increasing physical activity with mHealth interventions, setting behavioral goals or enabling self-monitoring, as well as a combination thereof, might be beneficial. With increasing technological possibilities, interventions will become ever more complex, and it is crucial to report their content thoroughly. However, let us not forget: BCTs are not everything. It is also important that people like and use the interventions. Elements of gamification and appealing visual presentation could be considered to address this issue.

## Appendix

Multimedia Appendix 1 Description of included studies (population, country, duration, and outcomes).

Multimedia Appendix 2 Data spreadsheet and annotated analysis script.

## Abbreviations

BCT: behavior change technique
EPHPP: Effective Public Health Practice Project
ES: effect size
mHealth: mobile health
PA: physical activity
RCT: randomized controlled trial
